# Impact of the Affordable Care Act on Access to Care for US Adults With Diabetes, 2011–2012

**DOI:** 10.5888/pcd12.140431

**Published:** 2015-05-07

**Authors:** Derek S. Brown, Timothy D. McBride

**Affiliations:** Author Affiliation: Timothy D. McBride, Brown School, Washington University in St. Louis, St. Louis, Missouri.

## Abstract

**Introduction:**

Lack of health insurance is a barrier to medical care, which may increase the risk of diabetes complications and costs. The objective of this study was to assess the potential of the Affordable Care Act (ACA) of 2010 to improve diabetes care through increased health care access by comparing health care and health outcomes of insured and uninsured people with diabetes.

**Methods:**

We examined demographics, access to care, health care use, and health care expenditures of adults aged 19 to 64 years with diabetes by using the 2011 and 2012 Medical Expenditure Panel Survey. Bivariate descriptive statistics comparing insured and uninsured persons were evaluated separately by income above and below 138% of the federal poverty level (FPL), (a threshold for expanded Medicaid eligibility in select states under the ACA) using the *t* test and proportion and median tests.

**Results:**

Uninsured adults reported poorer access to care than insured adults, such as having a usual source of health care (69.0% vs 89.5% [**≤**138% FPL], 77.1% vs 94.6% [>138% FPL], both *P* < .001) and having lower rates of 6 key diabetes preventive care services (*P* ≤ .05). Insured adults with diabetes had significantly higher health care expenditures than uninsured adults ($13,706 vs $4,367, $10,838 vs $4,419, respectively, both *P* < .001).

**Conclusion:**

Uninsured adults with diabetes had less access to health care and lower levels of preventive care, health care use, and expenditures than insured adults. To the extent that the ACA increases access and coverage, uninsured people with diabetes are likely to significantly increase their health care use, which may lead to reduced incidence of diabetes complications and improved health.

## Introduction

In 2012, more than 29 million Americans were living with diagnosed diabetes (1). The serious health challenges facing people with diabetes include heart disease, stroke, hypertension, kidney disease, neuropathy, and blindness (2). Researchers estimate that the economic burden to society of diagnosed diabetes reached $245 billion in 2012 (3). Although private and public health insurance programs provide important access to health care for some people with diabetes, millions of working-age adults with diabetes lack health insurance (4). This suggests that a high proportion of the population with diabetes faces significant challenges in access to health care, which may lead to suboptimal care, increased rates of long-term complications, and greater health care expenditures.

The Affordable Care Act (ACA) of 2010 is designed to provide access to coverage for previously uninsured Americans. Adults with incomes below 138% of the federal poverty level (FPL) will gain access to Medicaid coverage in states that expand coverage (5) (28 states including the District of Columbia as of January 24, 2015). People with incomes above the poverty level in all states can obtain access to private insurance plans in health insurance “marketplaces.” In addition, premiums in these marketplaces are subsidized for people with household incomes between 100% and 399% of the FPL (6). An estimated 60% of the uninsured will obtain health insurance through one or the other of these 2 methods by 2019 (7). As of September 2014, ACA had reduced the number of uninsured by more than 9 million (8), although a separate breakdown for people with diabetes was not available.

Previous published work has shown that the uninsured face significant barriers to obtaining health care and face higher out-of-pocket health care costs than the insured (9). In addition, the uninsured can experience health problems as a result of the lack of access to medical care. Although much research has focused on the general uninsured population, few studies have focused on the population with diabetes. A study similar to ours focused on Medicaid and diabetes, although the authors used older data and did not include people with higher incomes (10), who are also affected by ACA. In addition, because health care reform, one of the most important social policy changes in the United States in decades, is now nearly fully implemented, no studies have taken a snapshot of the uninsured US population with diabetes and considered how their medical care may be changing under full implementation of ACA in 2014 and beyond.

The objective of this study was to gauge the potential impact of ACA on improving diabetes care through improved health care access by comparing health care and health outcomes of a large national sample of insured and uninsured adults with diabetes. Our results provide a straightforward comparison of the gap between the insured and uninsured before health care reform and insights about how indicators for these 2 groups may converge in coming years.

## Methods

To obtain the latest pre-ACA snapshot of the US population with diabetes, we pooled data from the 2 most recent years of the Medical Expenditure Panel Survey (MEPS), the 2011 and 2012 household component full-year consolidated data files (11,12). (This period is “pre-ACA” because major provisions were not effective until 2014, although limited features such as expanded coverage for young adults began in 2010.) MEPS is an ongoing set of surveys sponsored by the Agency for Healthcare Research and Quality (AHRQ) that collects nationally representative data on health services and expenditures of the noninstitutionalized civilian population. MEPS is constructed from a subsample of households participating in the National Health Interview Survey and uses a stratified random sample design and computer-assisted in-person interviews (13). MEPS is well suited for this analysis because it provides detailed information on health care use and expenditures and also includes survey responses, capturing data on items such as health care access. Other national surveys and claims data provide one or the other type of data but rarely both.

MEPS respondents are interviewed 3 times during a calendar year and asked several questions about health care use, insurance, expenditures, access to care, preventive care services, and chronic diseases. People with diabetes are asked about health outcomes and health care specific to diabetes. For many measures on the full-year consolidated file, MEPS combines data from each respondent’s multiple interviews to create 1 calendar year variable.

We restricted the study sample to adults aged 19 to 64 years who reported that they had been diagnosed with diabetes (most people over age 65 with diabetes are unaffected by ACA coverage provisions, because they are eligible for Medicare.) We stratified analysis by household income at less than 138% of the FPL (hereafter, low income) and greater than 138% of the FPL (hereafter, high income), because these are the eligibility limits for Medicaid in states choosing to expand coverage.

We used edited variables when possible, which are cleaned for consistency by AHRQ across the multiple survey rounds in MEPS. These variables included age, sex, race/ethnicity, education, census region, and health insurance status. We collapsed the level of detail on variables such as employment, because these measures serve only as controls intended to identify systematic differences between the sample groups and are not primary aspects of diabetes care or outcomes. All outcomes, which are measured over a period of time (employment, insurance, access to care, diabetes care, health care use, and health care expenditures), were limited to the current year, 2011 or 2012, for the corresponding MEPS sample. For ease of exposition, we created the following mutually exclusive groups for the previous 12 months out of the monthly MEPS insurance indicators in the following order: dual eligible (Medicaid and Medicare), Medicaid, Medicare, private insurance, TRICARE and Civilian Health and Medical Program of the Department of Veterans Affairs (CHAMPVA), and other public insurance. People with more than 1 insurance type were grouped into the first applicable type in the insurance indicator list.

Diabetes-specific measures were from the MEPS Diabetes Care Survey (DCS), a paper-and-pencil survey module administered to those reporting that they had ever been diagnosed with diabetes. The DCS includes self-report of the year of diagnosis, of ever having had diabetes complications of the eye or kidney, and of receiving 6 preventive care measures in the past year (hemoglobin A1c blood test, feet checked for sores or irritations, dilated eye examination, blood cholesterol check, influenza vaccination, and blood pressure checks). We calculated the years since diabetes was first diagnosed as the difference between the respondent’s age at interview and the age at diagnosis. We used the DCS-specific survey weight when reporting DCS measures.

For physical health and comorbidities, we analyzed self-reported body mass index (BMI) (kg in weight/m^2^ in height) and the prevalence of all adult priority conditions, as defined by AHRQ (14). In addition to diabetes, the conditions are hypertension, heart disease (coronary heart disease, angina, myocardial infarction, other unspecified heart disease), stroke, emphysema, chronic bronchitis, high cholesterol, cancer, joint pain, arthritis, and asthma. Several of these are either linked to diabetes as complications associated with diabetes management and duration; others are risk factors that present additional challenges to proper diabetes care.

We included 3 broad measures of access, measured as binary indicators. All respondents were asked if they had a usual source of medical care in the past year. Respondents were also asked if they were ever unable to access necessary medical care in the past year and if they were unable to get necessary prescription medications. For health care use in the past year, we studied the number of physician or clinic office visits and total number of prescriptions. We included binary indicators for any emergency department visit and any inpatient hospital nights, because these services are used infrequently and their statistical distribution is skewed. We examined total health care expenditures, out-of-pocket health care expenditures, and prescription drug expenditures, which included diabetes supplies.

Survey-specific procedures in Stata 13.1 (StataCorp LP) with weighted analyses and analytic subpopulations were used. To pool the 2011–12 data, we halved the survey weights so that the results equally represented people with diabetes for 2011 and 2012. Differences between insured and uninsured people were examined separately by income group using means, proportions, and crosstabs. Tests of significance were computed using survey-specific *t* tests, proportions, and χ^2^ tests. We also computed medians of health care expenditure and use data and tested differences by using the nonparametric k-sample test on equality of medians.

## Results

We estimated from MEPS that from 2011 through 2012, more than 13 million adults in the United States aged 19 to 64 years were living with diagnosed diabetes, and nearly 2 million of them lacked health insurance (Table 1). The prevalence of diabetes ranged from 4.8% among the uninsured with incomes above 138% of the FPL to 10.5% among the insured with incomes at or below 138% of the FPL (Table 1); in both income groups, insured persons were more likely to have diabetes than uninsured persons (*P* < .001).

**Table 1:** Health and Demographic Characteristics of US Adults With Diabetes Aged 19 to 64 Years, Medical Expenditure Panel Survey 2011 and 2012

Characteristic^a^	≤138% Federal Poverty Level			>138% Federal Poverty Level			
	Uninsured^b^ (95% CI)	Insured^b^ (95% CI)	*P* Value^c^	Uninsured^b^ (95% CI)		Insured^b^ (95% CI)	*P* Value^c^
Diabetes prevalence	5.6 (4.8–6.6)	10.5 (9.3–11.6)	<.001	4.8 (4.0–5.6)		6.7 (6.1–7.2)	<.001
Unweighted sample (n = 1,568)	325	774	—	318		1791	—
Weighted sample (n = 13,084,968)	770,404	2,529,894	—	1,024,650		8,704,030	—
**Demographic characteristic**							
Age (mean), y	49.8 (48.4–51.1)	50.8 (49.8–51.9)	.19		51.8 (50.2–53.5)	52.3 (51.6–53.0)	.62
Female	51.1 (43.6–58.6)	53.9 (49.2–58.7)	.52		43.6 (35.0–52.2)	.464	.57
Race/ethnicity nonwhite	68.1 (58.9–77.3)	53.2 (46.3–60.1)	.007		54.7 (45.2–64.3)	35.4 (31.8–39.0)	<.001
Years of education, mean	11.3 (10.7–11.9)	11.6 (11.3–12.0)	.28		11.7(11.1–12.3)	13.5(13.4–13.7)	<.001
Ever employed in calendar year 2011 or 2012	42.7 (35.1–50.3)	26.6 (21.8–31.5)	<.001		69.6 (61.7–77.6)	73.8 (71.0–76.5)	.33
Census region: Northeast	8.1 (4.4–14.7)	20.8 (15.7–27.0)	.002		14.4 (8.7–22.7)	14.6 (12.0–17.7)	.22
Census region: Midwest	18.7 (12.7–26.6)	18.4 (14.6–22.8)			16.7 (11.2–24.1)	23.8 (19.5–28.7)	
Census region: South	55.8 (46.7–64.5)	38.7 (33.0–44.7)			44.0 (35.5–53.0)	43.2 (39.2–47.3)	
Census region: West	17.4 (11.4–25.7)	22.1 (17.3–27.9)			24.9 (18.3–32.9)	18.4 (15.6–21.4)	
Urban/metropolitan statistical area	82.8 (76.3–89.2)	76.7 (70.1–83.4)	.15		87.7 (81.6–93.7)	83.0 (79.3–86.6)	.16
**Type of health insurance in 2011 or 2012**							
Medicaid	—	47.8 (41.4–54.4)	<.001		—	6.9 (5.6–8.6)	<.001
Medicare	—	12.9 (10.1–16.4)			—	9.0 (7.2–11.2)	
Both Medicaid and Medicare	—	17.1 (13.2–21.8)			—	2.6 (1.8–3.8)	
Private insurance	—	18.1 (13.7–23.5)			—	78.8 (75.8–81.4)	
TRICARE/CHAMPVA^d^	—	3.1 (15.1–6.2)			—	1.9 (1.3–2.9)	
Other public insurance	—	1.0 (0.4–2.4)			—	0.8 (0.4–1.7)	
**Diabetes and associated conditions **							
Age of onset, y	40.3 (38.0–42.6)	39.6 (37.9–41.2)	.58		41.8 (39.4–44.1)	41.9 (40.8–42.9)	.95
Years since diagnosis	9.5 (7.6–11.5)	11.1 (9.8–12.3)	.14		10.2 (8.2–12.2)	10.5 (9.6–11.4)	.82
Kidney problems	12.9 (7.4–18.3)	15.0 (11.2–18.8)	.48		12.5 (6.8–18.2)	7.9 (6.2–9.7)	.12
Eye problems	24.9 (17.6–32.2)	28.8 (24.1–33.5)	.38		20.8 (13.8–27.8)	16.7 (14.0–19.3)	.26
**Physical health and comorbidities**							
BMI (kg/m^2^)	32.7 (31.4–33.9)	33.8 (33.0–34.7)	.14		31.5 (30.4–32.6)	33.5 (32.8–34.2)	.002
BMI overweight (25.0–29.9)	28.5 (21.7–36.3)	22.4 (18.3–27.1)	.17		33.8 (26.8–41.5)	24.7 (21.8–28.0)	.02
BMI obese I (30–34.9)	26.5 (20.5–33.5)	26.9 (22.5–31.9)	.91		29.7 (23.5–36.8)	28.7 (25.9–31.7)	.78
BMI obese II/III^e^ (≥35.0))	34.4 (27.0–42.5)	38.9 (33.4–44.8)	.34		24.3 (17.8–32.4)	35.4 (31.7–39.3)	.01
High blood pressure/hypertension	72.0 (65.1–78.9)	76.3 (71.8–80.7)	.31		72.9 (65.8–80.0)	68.7 (65.4–71.9)	.27
Coronary heart disease, angina, acute myocardial infarction, other heart disease	20.0 (12.9–27.1)	32.2 (26.9–37.4)	.007		22.0 (15.0–29.0)	21.9 (18.8–25.0)	.99
Stroke	4.5 (0.6–9.7)	13.0 (9.6–16.4)	.007		7.7 (2.5–12.8)	6.2 (4.7–7.7)	.60
Emphysema	1.2 (0.0–2.6)	6.2 (4.7–7.7)	.002		1.8 (0.0–4.1)	1.8 (1.0–2.6)	.97
Chronic bronchitis	4.5 (1.5–7.6)	12.4 (8.1–16.7)	.004		8.0 (2.9–13.1)	5.5 (3.7–7.2)	.36
High blood cholesterol	60.5 (52.1–69.0)	68.8 (64.3–73.3)	.09		53.8 (44.5–63.1)	67.3 (64.3–70.4)	.008
Cancer	7.5 (3.5–11.4)	11.6 (8.6–14.6)	.10		9.5 (2.4–16.6)	13.0 (10.3–15.7)	.39
Joint pain	57.2 (49.6–64.9)	71.7 (67.4–75.9)	.001		60.0 (51.6–68.3)	64.2 (60.7–67.6)	.36
Arthritis	36.5 (29.0–44.0)	54.6 (48.7–60.5)	.001		30.0 (21.9–38.2)	39.8 (36.1–43.4)	.04
Asthma	9.9 (5.3–14.5)	26.5 (21.3–31.7)	<.001		10.1 (4.4–15.8)	11.6 (9.7–13.6)	.62
Self-rated general health as fair or poor versus excellent, very good, or good	56.9 (48.9–64.8)	59.2 (53.8–64.6)	.64		40.0 (32.5–47.6)	30.2 (27.2–33.2)	.02

Abbreviations: BMI, body mass index; CI, confidence interval.

a Values are percentages unless otherwise noted.

b Tests of insurance status are between >138% federal poverty level insured and ≤138% federal poverty level. All other tests are by insurance status within income groups. All results were weighted to be nationally representative.

c Tests of significance were computed by using survey-specific *t* tests for continuous variables, proportions tests for binary variables, and χ^2^ tests for proportions.

d TRICARE/CHAMPVA is coverage for military families and dependents through the TRICARE system and Civilian Health and Medical Program of the Department of Veterans Affairs (CHAMPVA).

e Obese class II = BMI 35.0–39.9; obese class III = BMI ≥40.

Differences by insurance status suggest some patterns that may be related to both the likelihood of having insurance and the prevalence of diabetes. In both income groups, uninsured persons were more likely to be nonwhite (*P* = .007, low income; *P* < .001, high income). Among the low-income groups, uninsured people with diabetes (42.7%) were more likely than the insured to be employed (26.5%) (*P* < .001). Significant regional differences by insurance status were also apparent among the low-income groups (*P* = .002); more than 55% of low-income uninsured adults and only 39% of insured adults resided in states in the southern census region.

High-income, insured adults with diabetes had a higher average BMI than uninsured adults (33.5 vs 31.5, *P* = .002); this was also the case with overweight adults (*P* = .02) and those with high levels of morbid obesity (class II/III, *P* = .01). Low-income insured adults had significantly higher rates of 7 chronic conditions (heart disease, stroke, emphysema, bronchitis, joint pain, arthritis, asthma) than those without insurance (all *P* < .01 or smaller), and high-income people had higher rates of 2 conditions (high cholesterol and arthritis) (both *P* < .05).

Significant differences in health care access were seen in both income groups, both in having a usual source of care (*P* < .001) and being unable to access necessary health care (*P* < .001). Low-income people were also much more likely to report that they were unable to get necessary prescription medications (*P* = .002). Significant differences by insurance status for all 6 recommended diabetes preventive care services (ie, Hemoglobin A1c [HbA1c] test, foot examination, eye examination, blood cholesterol check, influenza vaccine, and blood pressure check) were found across income groups (*P* ≤ .05).

In both income groups, insured adults with diabetes were much more likely to have used medical services in the past year than those without health insurance (Table 2). For instance, in the low-income group, the mean number of annual office visits among the insured was nearly triple the mean number among the uninsured (*P* < .001) and nearly double that among the high-income group (*P* < .001). The mean number and the median number of prescriptions were also substantially higher among the insured in both income groups (*P* < .001). The likelihood of using emergency department services (*P* = .001) or having inpatient hospital nights (*P* < .001) in the past year was significantly greater (*P* < .001) among the low-income group than the high-income group.

**Table 2:** Health Care Access, Use, and Expenditures of US Adults Aged 19 to 64 Years With Diabetes, Medical Expenditure Panel Survey 2011 and 2012

Measure^a^	≤138% Federal Poverty Level			>138% Federal Poverty Level		
	Uninsured (95% CI)	Insured (95% CI)	*P* Value^b^	Uninsured (95% CI)	Insured (95% CI)	*P* Value^b^
**Access to and use of health care in 2011 or 2012**						
Had a usual source of care	69.0 (60.6–77.5)	89.5 (86.3–92.7)	<.001	77.1 (71.0–83.1)	94.6 (93.1–96.1)	<.001
Unable to access necessary medical care	18.5 (12.7–24.4)	6.5 (4.1–8.8)	<.001	13.8 (7.9–19.6)	2.5 (1.6–3.5)	<.001
Unable to get necessary prescription medications	17.6 (11.1–24.2)	6.4 (3.9–8.9)	.002	9.0 (3.9–14.1)	4.0 (2.7–5.3)	.06
HbA1c test	83.4 (76.4–91.3)	95.6 (93.2–98.0)	.003	86.6 (79.2–94.0)	97.5 (96.5–98.6)	.004
Feet checked	48.5 (38.9–58.0)	67.0 (62.7–71.3)	<.001	49.9 (40.1–59.7)	69.6 (66.6–72.7)	<.001
Eye examination	35.9 (27.5–44.2)	58.1 (52.7–63.3)	<.001	38.8 (30.2–47.4)	65.6 (62.1–69.1)	<.001
Cholesterol check	66.2 (58.5–73.9)	77.4 (73.2–81.6)	.015	69.5 (62.4–76.6)	88.1 (85.8–90.2)	<.001
Influenza vaccine	32.4 (25.3–39.6)	57.2 (51.4–62.9)	<.001	37.8 (28.1–47.5)	61.8 (58.6–64.9)	<.001
Blood pressure check	84.4 (79.3–89.4)	96.5 (94.4–98.6)	<.001	90.3( 86.7–94.0)	98.1 (97.4–98.8)	<.001
Office visits, mean no.	4.1 (3.0–5.2)	11.0 (8.8–13.1)	<.001	4.6 (3.5–5.6)	8.8 (7.9–9.7)	<.001
Office visits, median no.	2	5	<.001	3	5	<.001
Total prescription fills in year, mean	23.4 (18.7–28.0)	51.3 (43.9–58.7)	<.001	23.5 (18.4–28.6)	36.2 (34.0–38.6)	<.001
Total prescription fills in year, median	17	37	<.001	15	25	<.001
Any emergency department visit	19.3 (14.7–24.0)	33.3 (28.3–38.2)	<.001	22.9 (16.4–29.4)	21.9 (19.3–24.6)	.79
Any inpatient nights	8.9 (5.2–12.6)	25.6 (20.8–30.3)	<.001	10.9 (5.7–16.1)	14.7 (12.3–17.1)	.22
**Expenditures in 2011 or 2012, $^c^**						
Total, mean	4,367 (2,558–6,176)	13,706 (11,514–15,897)	<.001	4,419 (2,891–5,946)	10,838 (9,796–11,879)	<.001
Total, median	1,297	6,382	<.001	1,483	4,767	<.001
Out-of-pocket, mean	1,177 (837–1,516)	755 (620–889)	.021	1,490 (994–1,985)	1,288 (1,175–1,401)	.43
Out-of-pocket, median	432	225	.005	795	808	.09
Prescription drugs and diabetes supplies, mean	1,194 (857–1,530)	4,296 (3,295–5,298)	<.001	1,492 (946–2,037)	3,414 (3,056–3,771)	<.001
Prescription drugs and diabetes supplies, median	355	1,894	<.001	346	1,744	<.001

Abbreviation: CI, confidence interval.

a Values are percentages unless otherwise stated.

b Tests of significance were computed using survey-specific *t* tests for continuous variables, proportions tests for binary variables, and nonparametric *k*-sample tests for medians. All results were weighted to be nationally representative.

c Expenditures are as reported in current-year dollars. Measures refer to the full calendar year for respondents in either the 2011 or 2012 household component full-year consolidated Medical Expenditure Panel Survey data files. Samples were pooled as described in the methods section.

Differences in health care use and differences in expenditures between the insured and uninsured in both income groups were large. Mean total expenditures were much greater among the insured, which probably reflects greater access to health care: nearly $6,400 higher for those with incomes above 138% of the FPL (*P* < .001) and more than $9,300 higher for those with incomes at or below138% FPL (*P* < .001). Median differences were slightly smaller but still significant for both groups (*P* < .001). Out-of-pocket expenditures were higher among the uninsured only in the low-income group. Prescription drug expenditures were a significant driver of total expenses and were much greater among the insured than uninsured for both income groups (*P* < .001).

## Discussion

Our findings showed that from 2011 through 2012, shortly after passage of ACA, nearly 2 million working-age adults with diabetes lacked health insurance. We also showed that access to care was a significant barrier among this population and that proper diabetes care lagged among the insured on all indicators. Thus, the potential of ACA to improve health and health care for people with diabetes appears to be large. If the health care patterns of the uninsured in 2011–12 move toward those of the insured, our results suggest that expanded insurance coverage will likely increase health care costs in the short-term for people with diabetes. Although long-term effects were beyond the scope of our research, it is possible that these may be more favorable for health outcomes and expenditures. For example, a recent study found that weight loss among people with diabetes reduced health expenditures over 10 years (15), and other health care interventions have also been shown to reduce the burden of diabetes (16). Counterbalancing these findings are the findings from the Oregon Health Insurance Experiment (17), in which newly acquired Medicaid did not significantly reduce average HbA1c during the first 2 years of coverage. Our findings are consistent with that study in that we found higher rates of diagnosed diabetes and greater use of prescription medications among the insured than the uninsured samples.

Our findings showed that uninsured adults with diabetes undergo different patterns of care than those with health insurance. For instance, in our study the uninsured were much less likely to obtain prescriptions, make office visits to physicians, and to have a usual source of care. Rates of current multiple disorders, several of which are associated with diabetes, were greater among the insured than the uninsured in both income groups; differences were significant for 6 conditions among the low-income sample and for 2 conditions among the high-income sample. Possible explanations include self-selection, in which those who have a greater need for care seek insurance voluntarily, or improved access, in which the insured are diagnosed for these conditions at higher rates (4). The percentage of respondents reporting fair or poor health was nearly 10 points higher among the high-income uninsured group (*P* = .02), possibly supporting an access theory, although no significant differences were seen among the low-income group.

The observed disconnect between patterns of care provided and need for care suggests that as uninsured adults with diabetes obtain new health insurance under full implementation of ACA in 2014 and beyond, we should expect their use of medical care to increase significantly — more office visits, prescriptions, and use of inpatient care. Our results distinguish between people with incomes below 138% of the FPL who will become eligible for Medicaid (in states expanding Medicaid) and those people with incomes above 138% of the FPL; results also demonstrate that health care use should increase for both groups, although the gap in services is larger among low-income persons, suggesting greater potential demand among low-income persons. However, many other barriers to good diabetes care exist, besides health insurance, that are not directly targeted by ACA, including education, literacy, language, attitudes, beliefs, and social support (18).

Given the longstanding finding (19) that the uninsured face difficulties obtaining access to a usual source of health care, it is not surprising that uninsured adults with diabetes in both the high- and low-income groups we studied were significantly less likely to report having a usual source of care (*P* < .001). Greater total expenditures among the insured probably reflect access, or the ease of obtaining necessary medical care. Early observations in the Oregon studies indicate similar trends (20). The fact that our findings show that inpatient and emergency department services were greater among the insured parallels findings in Oregon (21) and recent qualitative evidence (22). Furthermore, among those with Medicaid, access to primary care providers may be limited in some areas, given low provider reimbursement rates.

Our results should be interpreted with caution. First, our study compares descriptive results. A more detailed comparison could use multivariate analysis to control for associations between variables presented here. The descriptive findings here serve as a starting point and guide for future research.

Second, our results are based on cross-sectional data, so we cannot assign causation to the differences in health status, outcomes, and health care use, access, or expenditures between uninsured and insured people. People with health insurance are likely to be different from those without health insurance in some important ways, both observable and unobservable. Multivariate models can control for the observables, although some of the differences we found probably reflect both observable and unobservable factors. Our goal with this analysis was not to estimate causal inference. Rather, we sought to address the research gap in the literature on differences between the uninsured and insured population of people with diabetes, above and below the average Medicaid eligibility cutoff of 138% FPL, by assessing numerous health outcomes and health care access and use measures. The fact that our demographic indicators show a high level of Medicaid use by the income group at or below 138% FPL and a high level of private insurance by the over-138% FPL group supports the value of such a comparison. That is, the uninsured in each group who become insured after ACA are likely to receive most of their coverage through the dominant form of insurance for that income group (Medicaid for low income, private insurance for high income). Although we expect some changes in patterns of health care and health care use to result from changes in coverage, measurement of the actual effects will need to be conducted in future research studies after additional years of post-ACA implementation data are available. We also see relatively few differences in demographics between the insured and uninsured, except for nonwhite race/ethnicity and region, which is probably due to the more restrictive Medicaid income criteria for low-income adults in Southern states.

Third, the study is limited to adults aged 19 to 64 years, so the findings here cannot be extended to children or the elderly, although they are less directly targeted by ACA. Fourth, the MEPS measure of the diabetes population reflects currently diagnosed adults. The population with undiagnosed diabetes is sizable (4), and some of these people may be newly diagnosed under ACA as a result of improved health care access. On the one hand, the highest rate of undiagnosed diabetes is found among the uninsured (4), so our findings may be viewed as underestimates of the potential number of uninsured people with diabetes who could be assisted by implementation of ACA. We could not identify adults in the diabetes population who may or will not be eligible for coverage under ACA (noncitizens or persons living in states that will not expand Medicaid); this nonidentification would overstate the number of uninsured people with diabetes who could be assisted by implementation of the ACA.
